# Lenvatinib treatment for thyroid cancer in COVID era: safety in a patient with lung metastases and SARS-CoV-2 infection

**DOI:** 10.1097/CAD.0000000000001097

**Published:** 2021-06-25

**Authors:** Pietro Locantore, Valeria Del Gatto, Andrea Corsello, Alfredo Pontecorvi

**Affiliations:** Unit of Endocrinology, Fondazione Policlinico Gemelli – IRCCS, Università Cattolica del Sacro Cuore, Rome

**Keywords:** anti-VEGFR receptor, case report, coronavirus disease 2019, lenvatinib, thyroid cancer

## Abstract

During the coronavirus disease 2019 (COVID-19) pandemic, clinicians are required to manage patient care for pre-existing conditions. Currently, there are no clear indications regarding the management of lenvatinib-treated patients for radioiodine-refractory thyroid cancer and severe acute respiratory syndrome coronavirus 2 (SARS-CoV-2) infection. A 74-year-old male patient was treated with lenvatinib since March 2019, with disease recurrence in the thyroid bed and bilateral multiple lung metastases. The patient partially responded to treatment, with reduction in lung metastases. In September 2019, the patient tested positive for SARS-CoV-2 and isolated at home. Initially asymptomatic, the patient developed mild symptoms. Lenvatinib treatment continued with daily monitoring of vital signs. After telemedicine consultation of patient’s clinical condition, severity of symptoms was low. He tested negative for SARS-CoV-2 21 days after testing positive. The patient received the full course of lenvatinib treatment. This is the first reported case of a lenvatinib-treated patient who developed COVID-19 and could continue treatment. Despite concerns over COVID-19, clinicians should not overlook treatment of pre-existing diseases or discontinue treatment, particularly for cancer. Clinicians should evaluate a patient’s history and clinical presentation, monitoring the patient to reduce the development of complications in high-risk settings, avoiding treatment discontinuation.

## Introduction

Severe acute respiratory syndrome coronavirus 2 (SARS-CoV-2) and coronavirus disease 2019 (COVID-19) have emerged as a global pandemic. Patients with either a history of or active cancer may be at an increased risk of contracting the virus and developing complications [1]. Patients on oral chemotherapy with a history of lung disease are at particular risk, due to intercurrent disease potentially worsening respiratory function. It is paramount that cancer treatment continues whilst protecting patients against the virus. The aim of this case report is to determine the safety profile of continuing lenvatinib treatment for patients with radioiodine refractory (RR) advanced differentiated thyroid cancer (DTC), given that COVID-19 may worsen respiratory function.

Advanced DTC is treated by total or near-total thyroidectomy, followed, where necessary, by radioiodine (^131^I) and thyroid hormone suppressive therapy [2]. However, some patients are resistant to ^131^I, and cytotoxic chemotherapy is not very effective in patients with metastatic RR-DTC. Alternatively, lenvatinib, an oral multikinase inhibitor is a novel therapy to manage DTC [3].

## Case presentation

We present a case of a 74-year-old male patient treated with lenvatinib for advanced RR-DTC, since March 2019, with disease recurrence in the thyroid bed and bilateral multiple lung metastases (maximum diameter 7.4 × 5.7 cm).

He started treatment at 24 mg/day lenvatinib; however, following weight loss and nausea in September 2019, the dose was reduced to 18 mg/day. He showed partial response to treatment, with a progressive reduction of lung metastases (actual maximum diameter 6.5 × 3.2 cm).

Whilst on treatment in September 2020, the patient tested positive for SARS-CoV-2 via a nasopharyngeal swab. The patient isolated at home and was initially asymptomatic. After a few days, he developed mild symptoms (cough, diarrhea, and worsening asthenia), but never experienced anosmia or fever. We decided not to discontinue lenvatinib treatment and daily monitoring of vital signs was performed, including blood pressure, body temperature, and oxygen saturation. The evaluation of adverse events (AEs) and the patient’s clinical condition was carried out by telemedicine. Due to the low severity of symptoms, chest imaging was not performed. The patient performed a new nasopharyngeal swab 21 days after the detection of SARS-CoV-2 and tested negative. Despite our concerns, we observed no severe respiratory, gastrointestinal, or hematopoietic complications, and the patient needed neither specific therapy for COVID-19 nor lenvatinib interruption or discontinuation for the full course of the intercurrent disease.

## Discussion

Patients with cancer are typically older with more comorbidities and may be immunocompromised by treatment or through the nature of cancer [1]. Patients with cancer have an increased risk for COVID-19 related morbidity and mortality, regardless of whether they have active cancer or are being treated [1]. Careful monitoring of both COVID-19 symptoms and anti-cancer treatment associated AEs are important for assessing treatment continuation. To date, this is the first reported case of a lenvatinib-treated patient who developed COVID-19, with the patient able to continue treatment without experiencing any additional AEs. As data are limited, this report is an important indicator of the safety of continuing lenvatinib treatment during the COVID-19 pandemic and could be more widely generalized to patients with COVID-19 infections for other cancer types receiving anticancer treatments.

Patients with RR-DTC treated with lenvatinib were enrolled in an Italian expanded access program to characterize the safety profile [4]. All patients experienced at least one AE, the most frequent AEs reported were hypertension (80.5%), fatigue (58.3%), diarrhea (36.1%), stomatitis (33.3%), hand-foot syndrome (33.3%), and weight loss (30.5%) [4].

Despite the complications of COVID-19 and the increased risk of mortality and morbidity for patients with cancer and SARS-CoV-2, continuing lenvatinib treatment should be favored over treatment discontinuation, given the treatment benefits. In a phase 3 trial, lenvatinib treatment significantly improved progression-free survival (PFS) for patients with RR-DTC, with a 14.6-month longer median PFS vs. patients receiving placebo (*P* < 0.001) (5). Lenvatinib significantly improved response rate (64.7% in the lenvatinib group vs. 1.5% in the placebo group, *P* < 0.001) [5]. In a post hoc analysis performed on patients enrolled in the SELECT study, higher rates of dose interruption or dose reduction had a negative impact on PFS [6].

In the present case, we could hypothesize that effective monitoring of a patient’s AEs and clinical presentation is important in deciding if lenvatinib treatment should continue for patients testing positive for SARS-CoV-2. The continuation of treatment for patients with cancer is crucial for disease management. Careful monitoring of patients by clinicians could ensure lenvatinib treatment continues whilst managing the complications in high-risk settings due to the COVID-19 pandemic [7]. In summary, this case report demonstrates that lenvatinib treatment can continue if patients are carefully monitored for COVID-19-associated complications. Studies with larger samples and longer follow-up periods are required to determine the safety of continuing cancer treatment for patients with cancer and COVID-19.

## Acknowledgements

The authors confirm that the research meets the ethics guidelines, including adherence to the legal requirements of the country where the study was performed.

All authors have equally contributed. All authors have read and approved the article.

Written consent for publication was obtained from the study participant.

The dataset used during the current study is available from the corresponding author on reasonable request.

### Conflicts of interest

There are no conflicts of interest.
